# The Prognosis of Cancer

**Published:** 1910-12-10

**Authors:** 

**Affiliations:** Professor of the Faculty of Medicine, Member of the Academy of Medicine, Surgeon to the Hôspital de la Charité, Paris.


### Hospital Clinics.

### THE PROGNOSIS OF CANCER.

Lecture by DR. RECLUS, Professor of the Faculty of Medicine, Member of the Academy of Medicine, Surgeon to the Hôspital de la Charité, Paris.

During the course of the last fifteen or twenty years almost every day some new treatment of cancer has cropped up. We have seen successively radiumtherapy, radiotherapy, high-frequency currents, salts of soda, potash and quinine, and a number of analogous substances advocated, not to mention an infinite variety of serums, curative or preventive. Singularly enough, not one of these methods but seemed at first to give good results, and, if not absolutely to cure, at any rate to produce very marked amelioration in the state of the patients. Surgeons in every country pounced upon these vaunted methods; but less than a decade has always sufficed to shatter the hopes entertained. At first sight we can hardly understand how it is that men of science, honourable and not devoid of critical acumen, should allow themselves to be taken in by these deceptive discoveries and to publish accounts of very ephemeral successes. We must not forget, however, that cancerous patients are generally treated without very much conviction, and are very often abandoned to the regular use of morphine. One day a saviour comes, he communicates his own confidence and enthusiasm to the patient, hope and the desire to live are renewed, sleep returns, the appetite and strength are increased, and both physician and patient are delighted and proclaim as incontestable a cure which in reality is only imaginary. I remember quite well, at the Society of Surgery, under the presidency of our regretted colleague Bouilly, an inventor of a new serum bringing an intelligent and cultured patient, who declared that he was completely and definitely cured of a cancer of the tongue, whereas the tissues still presented the rigidity of cardboard.

### Malignity.

The real reason of our mistakes is our ignorance of the course and evolution of cancer. As soon as an histological examination, with or without the aid of clinical symptoms, has allowed us to make the diagnosis of malignant growth, the death-warrant of the patient is signed without any possibility of appeal. We conclude from an identical structure an identical termination, and because the anatomical elements are the same and are grouped in a similar tissue in each of the tumours, the bearer of the one will have the same fate as the bearer of the other. We forget that above the question of structure there is a whole series of unknown conditions which constitute what, in our ignorance, we call *malignity*—certain growths presenting a most rapid course and killing in the space of a few months, whilst others take years to do so. If then, by some happy chance, a physician comes across one of the latter variety, we can easily understand how he may, in all good faith, entertain very great hope and attribute any amelioration or slowness of development to the treatment employed. It is for this reason that I wish to speak to you to-day of the prognosis of cancer.

### Is Cancer Always Fatal?

Our treatises on general pathology tell us that the termination of cancer is always fatal, and nearly always very rapidly so. The first is only too true, and one might count on one's fingers the really authentic cases of spontaneous cure. Pre-antiseptic surgery used to speak of epithelial ulcerations being cured by erysipelas; but, as a matter of fact, cicatrisation did not last very long, and new indurations soon appeared in the tissues. Besides, these cases consisted of cancroids of the skin, for as regards the deep-seated cancers, of the breast for instance, they are still more open to dispute. I myself have seen, when quite a young student, an old relative of mine over ninety years of age whose breast, pectorals and ribs had been destroyed by a growth of fifty years' standing which had been considered to be a cancer by such eminent clinicians as Paul Broca, Follin, and Racle. At this period, however, histology was still in its childhood and such cases are always open to the accusation of erroneous diagnosis.

If the termination is always fatal, at any rate it is not always rapidly so; there are even certain cancers whose evolution is generally very slow. I have in mind those cancroid or rodent ulcerations of the skin, especially frequent in the cheek, forehead, and nose, and which may also be found on the limbs developing on some old ulcer or scar. Who has not come across some of these *croûtes des vieillards* lasting for years and years, scarcely spreading at all and without affecting the general state of health, the patients dying of some intercurrent affection?

### Some Illustrative Cases.

I have, together with Broca, attended one of our most distinguished literary men, who, although suffering from an epithelioma of the cheek, led for fourteen years a most active life. I have described another very remarkable variety of cancer with a very slow course, the "surface epithelioma of leukoplakial mucous membranes." A third variety consists of the *cancers en cuirasse*, which are rare, but not exceptional, and of which each of you, I am sure, has had the opportunity of observing examples. I have attended an old lady who, for a period of ten years, had both breasts like armourplate, and who died eventually of pulmonary complications due to the progressive contraction of the chest by the hardened tissues. In another case, when preparing to operate for strangulated umbilical hernia, I found the patient presented a cancer *en cuirasse* extending over the whole of the front of the chest wall; it had existed for more than fourteen years, and none of her relatives knew anything about it. She died only several years later. In both these cases I prescribed Fowler's solution, but, of course, I do not pretend that this drug had any retarding action on the course of the disease.

### Abdominal Cancer.

Certain visceral cancers are not incompatible with long survival; certain growths of the parotid, branchiomata of the neck, epithelial neoplasms of the bladder may remain quiescent for many years before assuming the rapid course of malignant tumours. Cancers of the kidney do not always have a rapid evolution. In conjunction with Professor Guyon I have attended, during the last twelve years, a professional man presenting a very large and deformed kidney, which gives rise every now and then to very serious hæmaturia, with an acute state of anæmia. He is still living, and does not suffer from cachexia. Professor Guyon has observed a similar, but even more remarkable, case—namely that of a Bordeaux magistrate, who lived for over nineteen years in spite of a very large kidney and frequent hæmaturia.

### Post-operative Prognosis.

I have spoken, up to the present, only of cancer abandoned to itself. I must now consider the prognosis of cancer after operation.

If all surgeons agree that excision is the best treatment of malignant growths, many are of opinion, and not without reason, that some form of post-operative medication may check and even prevent relapse. Some propose the use of certain preventive serums, others have recourse to radiotherapy applied either to the open wound left unsutured or to the skin of the region operated after closing up the wound. Well, we shall find here the same complexity and the same paradoxical evolution, the course of the disease being in one case retarded and in another precipitated, and that often quite independently of all clinical rules. It is very important to be acquainted with these facts when investigating or experimenting any new treatment, for otherwise we might be inclined to give our confidence to medications, in reality quite inefficacious, simply because after the operation there has been no relapse for a period of two or three years, whereas it is well known that this may be the case after simple excision without any after treatment whatever, such as radio or radium therapy, serums, etc. Without invoking certain conditions of age, temperament, heredity, diathesis, early and extensive excision, that mysterious factor "malignity" explains the differences in rapidity of evolution.

### Lingual Epithelioma.

What do we know, for instance, of epithelioma of the tongue? Leaving out of consideration the variety I have called "surface epitheliomata of leukoplakial membranes," I have no hesitation in declaring that cancer of the tongue is the worst of all, and in quoting Denonvilliers' dictum, "all ulcerated cancers of the tongue die within the year." Early and extensive operation is only too often ineffectual in spite of Poirier's affirmation to the contrary, and yet how many cases are there where, contrary to all expectation, there has been no recurrence?

Trelat mentions only thirteen cases in his statistics; but in more modern ones they are much more numerous. Personally I have observed some most remarkable cases. I have already described that of a jeweller whom I operated on in 1880, with the help of Dr. Brissaud. He was still alive in 1896, when I lost sight of him, but there was not at that time any sign of recurrence. In January 1886 I operated on a merchant of Versailles for a vegetating epithelioma of the anterior half of the tongue, with degenerated sub-maxillary glands. Immediately after the excision of the diseased part a rapid examination of the latter showed that the operation had been insufficient, so I seized the stump of the tongue with a Museux forceps and cut off another slice of the organ, in a rather haphazard fashion, I must admit. I feared a rapid recurrence very greatly, but it did not take place, and only a few days ago I received a letter from the patient saying that he is enjoying the best of health. Another extraordinary case is the following: In 1893 I operated, with the late Professor Verneuil, on a patient from Beauvais, presenting a large ulcerated cancer of the tongue with cervical adenopathy. The case seemed a very bad one; but in June 1906 the patient was still alive. He presented, at that time, an indurated patch on the stump of the tongue, and I was very much afraid of a relapse. In October of the same year the fear became almost a certainty and Poirier, who was consulted during my absence, advised immediate operation. I did not approve of the latter on account of the cerebral, cardiac, and pulmonary state of the patient. I saw the man again more than a year later, and the tumour had but so very slightly increased that I almost doubted whether there would be any relapse.

### Carcinoma Mammæ.

Cancer of the breast is so frequent and operations for the same so numerous that statistics run into hundreds of cases. Halstead's statistics are wonderful, and if adeno-carcinomata are really cancers, as they are considered to be in America, we see from these statistics that three years and more after the operation half the patients are still alive without relapse. Of course, this proportion is exceptional, and in fact it seems to be obtained only by Nathan Jacobson and Chadwick Olliver. Other American authors give results approaching more those obtained in France, and see only one-third of their patients survive the third year without recurrence. This, however, is still very fine and it seems to be demonstrated that their method of operating—namely, free excision of the skin of the affected region, removal of the pectoral muscles, clearing out systematically the axilla and the supra and infra clavicular regions —has improved the prognosis of surgical intervention in a remarkable degree. We have also made much progress in France, and, referring to my own cases, I note that since 1901 I have obtained better results. Before that date 28 per cent. of my cases survived three years and more after operation, whereas out of twenty-one cases I have operated on since, without following exactly Halstead's technique but still applying very free excision, I count six deaths, one of which was due to embolism two months after operation, and five from more or less rapid relapse. Of the fifteen that are left one only presents certain relapse, and two others are very suspicious, but the twelve others show no sign of it. As seven of the latter have not passed the three years' limit yet, I do not wish to attach more value to my statistics than they deserve. Hospital results seem also very good, but as I have not had the opportunity of seeing many of my patients after the operation I will not say anything about them here.

### The Fallacies of Observation.

The success of modern surgery in cancer of the breast is such that it renders it very difficult to appreciate correctly the results obtained either by post-operative radiotherapy or preventive injections of serum. It will be necessary to collect very extensive statistics, and the number of successes will have to be much greater if we are to be convinced of the efficacy of *x*-rays or of vaccines. The difficulties are all the greater that we come across now and then some really disconcerting figures. In Kinderdgy's work, which dates from a period when it was not usual to touch the pectorals nor to clear out the axilla, and when even the breast was only partially removed, out of a series of eighty-five cases 26 per cent. were successful, that is escaped relapse. And how strange is the evolution of some of the relapses? In November 1897 I saw a woman who had been operated on a few months previously by one of my old assistants; some little while before I saw her several large nodules had made their appearance in the cicatrix and extended up beyond the clavicle. Such rapid recurrence seemed to announce a very near end; well, one year later the lesions appeared to have regressed; they remained stationary during seven years, and it was only in 1905 that the patient, who had been well and active up till then, died of generalised growths. Now if we had used some serum or vaccine, how could we have escaped the temptation to affirm its efficacy in prolonging this woman's life?

### Uterine Cancer.

Twenty years ago, cancer of the womb was considered to be nearly always fatal in a very short lapse of time, and after operation patients as a rule led but a very miserable sort of life. Since that time the prognosis seems to be less sombre, and many gynæcologists have published more encouraging statistics. I can hardly understand, however, those of Knauer, quoted by Pozzi, who, in a series of 213 operated cases, counted as many as seventy-six, that is over 30 per cent., in which patients survived a minimum of five years. All I can say is that if there is no error in the figures there is certainly one in the diagnosis. Other foreign statistics give a proportion of successes in 10, 15, 20, and even 25 and 30 per cent. In France J. L. Faure has also obtained very good results, and he mentions five patients who are still living after from one and a half to three and a half years, and seven from four to five years.

### Rectal Cancer.

Excision of malignant growths of the rectum which, like operation for cancer of the tongue, breast and uterus, has been greatly improved in technique, still remains a very serious matter, and gives a shorter survival than in the above cases; it was even shorter twenty years ago. I remember, however, a rather remarkable case. When rectal cancers first began to be removed by Kraske's method I operated, with the help of my colleague, Dr. Sebileau, on a solicitor from the Gironde, and, up to the present moment, he is still quite well.

I do not know whether you have quite followed me in the course of this lecture, but, in studying the evolution of cancer in the organs which are most frequently attacked by it, I wished to show you that before operation its course was not always fatally progressive; it may remain quiescent for a long time; it may present a respite of several years' duration, and after operation, if the tumour has been thoroughly removed, a cure, we may almost say a definite cure, is obtained in one quarter, and perhaps in one-third, of the cases.

### What is the Prognosis?

The early and extensive excisions of modern surgery play a very important rôle in the incontestible improvement of all the statistics. We must not lose sight of these facts if we wish to judge equitably any new method of treatment—*x*-rays, high-frequency currents, sera, etc.

Before expressing any opinion we must demand very large statistics indeed. We must not forget either that we are still ignorant of the evolution of cancer, that there are some very contradictory series of cases, some cancers having a very rapid and others a very slow course. We still do not possess the slightest notion why, of two growths presenting the same structure, located in the same organ, in persons of the same sex and of the same age, one will have a slow course or will be cured by operation, whilst the other will have a fulminating course and kill the patient in spite of early and extensive operation. Our prognosis in each individual case must be extremely reserved. Remember my case of cancer of the tongue operated in 1886; the intervention was not an early one, the glands were already attacked, nor was it an extensive one, yet twenty-four years later the patient was still alive.

### Quasi-miraculous Cases.

In 1904 a patient consulted me for a tumour of the axilla which I considered as a gland with caseous degeneration. I removed it under local anæsthesia (stovaine) which in itself shows that the excision was strictly limited; in fact, I simply enucleated the tumour. The latter turned out to be a melanic epithelioma consecutive to a cutaneous nævus which had been destroyed a few years previously by the thermo-cautery and the scar of which had escaped my notice. I greatly feared a rapid recurrence of this, the most terrible variety of all cancers; well, there has been no recurrence up to the present.

Another extraordinary example is the following case. In 1888 I removed an epithelioma of the angle of the orbit. Relapse in 1889, operation; in 1890 a third and in 1891 a fourth operation. During the next four years the patient did very well, but in 1895 the state of matters became so bad, the disease so deep and extensive, that I refused a further intervention. Drs. Tillaux, Le Dentu, and Terrier, who were successively consulted, concurred with me and refused to operate. The patient and his physician, my friend Dr. Paulin-Moizard, insisted, however, so vehemently that at last I gave way. I cleared out the orbital cavity, removed a part of the frontal bone, exposed the dura mater, and attacked the ethmoid and sphenoid bones. The cicatrisation of this huge loss of substance took place quite normally, and a few days ago I saw my patient in splendid health and with no recurrence.

But, alas! to these quasi-miraculous cases most disastrous ones can be opposed. A minister plenipotentiary, still young, consulted me on his return from China for a tumour of the tongue. It was of recent date, as large as a sixpenny piece, only very slightly ulcerated, and the glands seemed quite normal. In this case operation was early and very extensive, and I had the right to look forward to a success. Well, in three months' time recurrence took place *in loco*, spread to the neighbouring tissues, gave rise to acute adenitis with suppuration, and the patient died in less than ten months.

Gentlemen, keep these examples, both good and bad, in your mind. Remember that up to the present early and extensive excision is the only method of checking the evolution of cancer. Remember that, thanks to a bolder and more precise technique, great progress has been made, but do not forget, on the other hand, that we know scarcely anything about cancer. We know nothing of its origin and nature, nothing of its probable course in any given case, nothing of its prognosis.

